# Special issue on knowledge mobilization: Pediatric pain

**DOI:** 10.1002/pne2.12040

**Published:** 2020-11-26

**Authors:** Kathryn A. Birnie, Denise Harrison

**Affiliations:** ^1^ Department of Anesthesiology, Perioperative and Pain Medicine Cumming School of Medicine University of Calgary Calgary AB Canada; ^2^ Alberta Children’s Hospital Research Institute University of Calgary Calgary AB Canada; ^3^ Solutions for Kids in Pain (SKIP) Halifax NS Canada; ^4^ Department of Nursing Faculty of Health Sciences Faculty of Medicine, Dentistry and Health Sciences University of Melbourne Parkville VIC Australia

There is a recognized gap between existing pediatric pain scientific knowledge and the awareness and application of that knowledge by health professionals, healthcare institutions, children living with pain and their families, and the general public.^1^ Solutions for Kids in Pain (SKIP; www.kidsinpain.ca) is pleased to partner with Paediatric and Neonatal Pain to introduce this second of two special issues focused on knowledge mobilization. SKIP is a knowledge mobilization network whose mission is to improve children's pain management by mobilizing evidence‐based solutions through coordination and collaboration. Knowledge mobilization connects research, policy, practice, and lived experience expertise (patients, families) to increase dissemination and implementation of research and evidence‐based knowledge across organizations or sectors with the ultimate goal of improved pain management for children.^2^, ^3^

The two journal special issues highlight initiatives aimed to move knowledge in pediatric and neonatal pain into practice, and into the hands of people who will apply this knowledge in their everyday lives. The first special issue focused on knowledge mobilization in neonatal pain (Part 1) (https://onlinelibrary.wiley.com/doi/full/10.1002/pne2.12039). This second issue focuses on knowledge mobilization in pediatric pain (Part 2). The collection of five papers in this special pediatric edition is notable for showing the scope of knowledge mobilization activities.

The first two papers showcase application of patient engagement, also called patient and public involvement, to engage the expertise of people with lived experience with chronic pain during childhood as partners to inform pediatric pain research and care. Latimer and colleagues (https://onlinelibrary.wiley.com/doi/10.1002/pne2.12024) use community‐based conversations to gather perspectives of Indigenous people regarding pain experience, care experiences, and strategies to improve healthcare encounters. Their study applies a Two‐Eyed Seeing approach, a term coined by Mi'kmaw Elders Albert and Murdena Marshall, that considers both Indigenous and Western knowledge. Hurtubise and her team (https://onlinelibrary.wiley.com/doi/10.1002/pne2.12018) introduce their innovative application of collaborative logic analysis methodology to guide multi‐stakeholder engagement in evaluation of an intensive interdisciplinary pain treatment program (IIPT). Their team engaged a 13‐member expert panel of adolescents with lived experience with chronic pain, their parents, health professionals, teachers, and health systems managers. Efforts by both of these teams are noteworthy for their high degree of stakeholder partnership to advance the science and practice of patient engagement in pediatric pain research.

The latter two papers focus on effective design and delivery of pain education to make it meaningful for children and nurses. Pate and his collaborators (https://onlinelibrary.wiley.com/doi/10.1002/pne2.12015) explore the benefit of animated videos to provide wide‐reaching rigorous pain science education to children and adolescents across online platforms (websites, YouTube, Facebook). The paper highlights the research team's successful partnership with industry to create and share high‐quality online content about pain for youth. Their call to action is paired with practical tips to guide clinicians and researchers in similar endeavors to create engaging evidence‐based pediatric pain education content. Twycross and colleagues (https://onlinelibrary.wiley.com/doi/full/10.1002/pne2.12037) address head on how traditional professional education that focuses primarily on pain neuroscience, assessment, and management has not led to improved pediatric pain care and outcomes for families. Rather, they introduce the theoretically based *Ways of Knowing Pain* model that articulates the need to move beyond empirical knowing about pain to integrate esthetic, ethical, personal, and emancipatory knowing about pain. A case study is used to describe what skills, teaching strategies, and outcomes can be applied to bring pain care to life for nurses, and likely other healthcare professionals, to improve pain care for children.

The final paper in this special issue authored by Coakley and Bujoreanu (https://onlinelibrary.wiley.com/doi/10.1002/pne2.12019) outlines the knowledge mobilization roadmap of the Comfort Ability Program, a one‐day pain management workshop for children and adolescents with chronic pain and their parents, from program conceptualization to implementation, evaluation, iterative innovation, and sustainability. Their thoughtful description of patient and family, healthcare provider and institutional‐level considerations and partnerships for successful program implementation can be a guide for other innovations in pediatric pain care looking to successfully scale‐up and spread across a wide network.

We are optimistic as we reflect over the compilation of 9 papers included in the two special issues on knowledge mobilization published in Paediatric and Neonatal Pain. It is clear that neonatal and pediatric pain researchers are beginning to recognize the importance and value of diverse partnerships with people with lived experience, patients, families, healthcare professionals, decision‐makers, and industry to more effectively and efficiently facilitate uptake of knowledge in pediatric pain. However, there are key areas of knowledge creation that are not represented here, including basic and experimental laboratory‐based science, as well as knowledge users, such as policymakers and patient organizations with whom knowledge mobilization efforts are warranted. Although people at particular risk for poor pain care, such as Indigenous children, are included here, there is extraordinary need for intentional efforts to partner in knowledge mobilization with more vulnerable groups who are underrepresented in pain research and who are less likely to be able to access pain care. As a community, we should not forget that the timely coming together of rigorous science and powerful public outcry were integral to the birth of our field of pediatric and neonatal pain.^4^ Partnership with people with lived experience in all research is critical for driving continued change toward improved and sustained pain management for children and their families.^1^, ^5^, ^6^ We are proud of these two special knowledge mobilization issues produced in partnership between SKIP and the journal. We hope that the research showcased here will result in more consistent use of best evidence and subsequently, improved outcomes for neonates, infants, children, adolescents, and their families.
